# Electron Acceptors With a Truxene Core and Perylene Diimide Branches for Organic Solar Cells: The Effect of Ring-Fusion

**DOI:** 10.3389/fchem.2018.00328

**Published:** 2018-09-04

**Authors:** Kaiwen Lin, Shiliang Wang, Zhenfeng Wang, Qingwu Yin, Xi Liu, Jianchao Jia, Xiao'e Jia, Peng Luo, Xiaofang Jiang, Chunhui Duan, Fei Huang, Yong Cao

**Affiliations:** State Key Laboratory of Luminescent Materials and Devices, Institute of Polymer Optoelectronic Materials and Devices, South China University of Technology, Guangzhou, China

**Keywords:** organic solar cells, star-shaped electron acceptors, truxene, perylene diimide, ring-fusion

## Abstract

In this work, a star-shaped planar acceptor named FTr-3PDI was synthesized via ring-fusion between truxene core and three bay-linked perylene diimide (PDI) branches. Compared to the unfused non-planar acceptor Tr-3PDI, FTr-3PDI exhibits better structural rigidity and planarity, as well as more effective conjugation between truxene core and PDI branches. As a result, FTr-3PDI shows up-shifted energy levels, enhanced light absorption coefficient, increased electron mobility, and more favorable phase separation morphology in bulk-heterojunction (BHJ) blend films as compared to Tr-3PDI. Consequently, FTr-3PDI afforded higher power conversion efficiency (PCE) in BHJ solar cells when blended with a polymer donor PTB7-Th. This work demonstrates that ring-fusion is a promising molecular design strategy to combine the merits of truxene and PDI for non-fullerene acceptors used in organic solar cells.

## Introduction

Recently, non-fullerene electron acceptors have received considerable attention in the community of organic solar cells (OSCs) due to their energy level tunability, intense optical absorption properties, and potential for low-cost and large-scale fabrication (Cheng et al., 2018; Hou et al., 2018; Yan et al., 2018). Among them, perylene diimide (PDI) derivatives are widely investigated in bulk-heterojunction (BHJ) OSCs because of their intense light absorption and high electron mobility (Zhan et al., 2007, 2011; Lin et al., 2014; Sun et al., 2015; Hendsbee et al., 2016; Liu J. et al., 2016; Liu Z. T. et al., 2016; Meng et al., 2016a). Despite these favorable properties, PDI monomer shows low device performance due to the formation of large aggregated nanostructure and undesired large crystalline domains caused by the large coplanar structure of PDI block, which hamper the exciton diffusion and separation process (Sharenko et al., 2013; Liu S. Y. et al., 2015). To overcome these drawbacks, an effective strategy is to develop non-coplanar PDI-based molecules via forming twisted intramolecular structures (Zhong et al., 2014, 2016; Lin et al., 2016; Zhang et al., 2016; Duan et al., 2017a; Liu X. et al., 2017; Liu et al., 2018). For example, a lot of star-shaped electron acceptors with PDI branches were reported recently based on this design guideline (Lin et al., 2014, 2016; Liu Y. H. et al., 2015; Lee et al., 2016; Duan et al., 2017a; Zhang A. D. et al., 2017). Although these star-shaped PDI electron acceptors can avoid forming large crystalline domains, their highly twisted architectures decrease the intermolecular contact and orbital overlapping between PDI π-planes, thus hampering electron hopping between molecules.

Therefore, the key point to develop high-performance PDI electron acceptors is to obtain a balance between highly twisted non-planar structures for forming proper phase separation in blend films and strong intermolecular interaction for supporting sufficient charge transport ability.

**Scheme 1:**
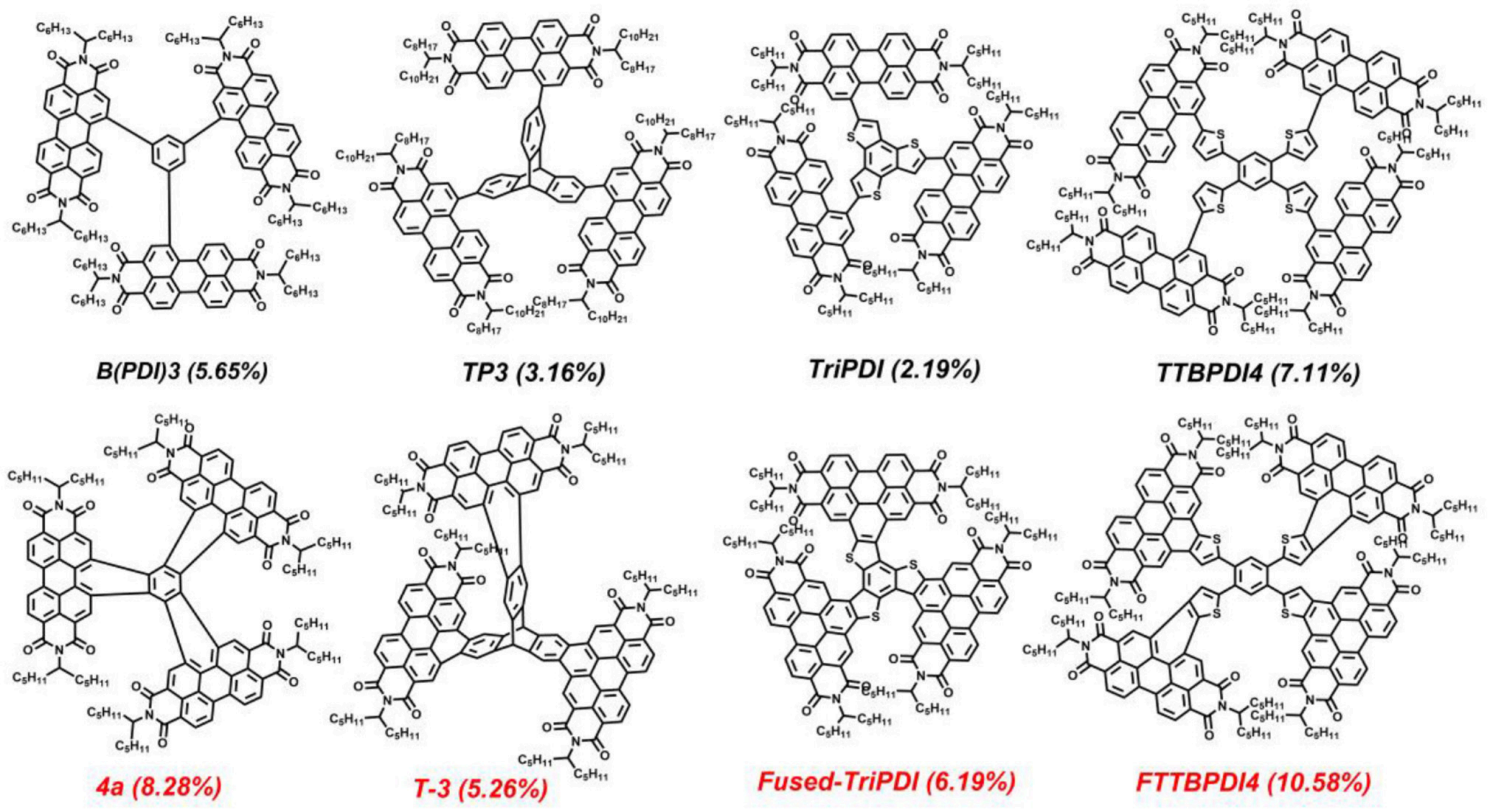
The reported representative star-shaped electron acceptors with PDI branches linked by carbon-carbon single bonds and adjoin benzene rings.

Recently, several studies showed that oxidative ring-fusion between the PDI branches and the central aromatic core of PDI-based molecules is an effective strategy to achieve such an exquisite balance (Hartnett et al., 2016; Meng et al., 2016b, 2017; Zhong et al., 2016; Wang B. et al., 2017; Zhang J. Q. et al., 2017). The resulting fused PDI molecules exhibit better structural rigidity and planarity, as well as more effective conjugation between the aromatic core and PDI branches. Meanwhile, the fused PDI molecules show stronger intermolecular π-π stacking and higher electron mobility. Moreover, these fused PDI acceptors can lead to desirable film morphology with proper domain size and high domain purity in BHJ blends when blended with donor polymers (Meng et al., 2016b, 2017; Wang B. et al., 2017; Zhang J. Q. et al., 2017). Therefore, the fused PDI acceptors display significantly improved photovoltaic performance compared to their carbon-carbon single bond connected counterparts (Scheme 1) (Li et al., 2016; Meng et al., 2016b, 2017; Liu X. F. et al., 2017; Wang B. et al., 2017; Zhang J. Q. et al., 2017). Actually, the best-performing OSCs based on PDI acceptors was achieved by a star-shaped fused PDI molecule named FTTB-PDI4, which afforded a power conversion efficiency (PCE) of 10.58% (Zhang J. Q. et al., 2017).

Among various central cores for star-shaped electron acceptors, truxene has been proved to be promising for constructing high-performance optoelectronic materials (Nielsen et al., 2013, 2014; Lin et al., 2018; Wu et al., 2018). The rigid coplanar structure and unique C_3h_ symmetry contribute to well-delocalized electronic structure in extended dimensionality for the resulting star-shaped conjugated molecules, which in turn result in strong light absorption and effective charge transport. Recently, Peng's group reported a state-of-the-art truxene-based electron acceptor for application in OSCs, which yielded impressive PCE exceeding 10% (Wu et al., 2018). These results suggested the promising prospect of truxene for constructing high-performance electron acceptors.

**Scheme 2:**
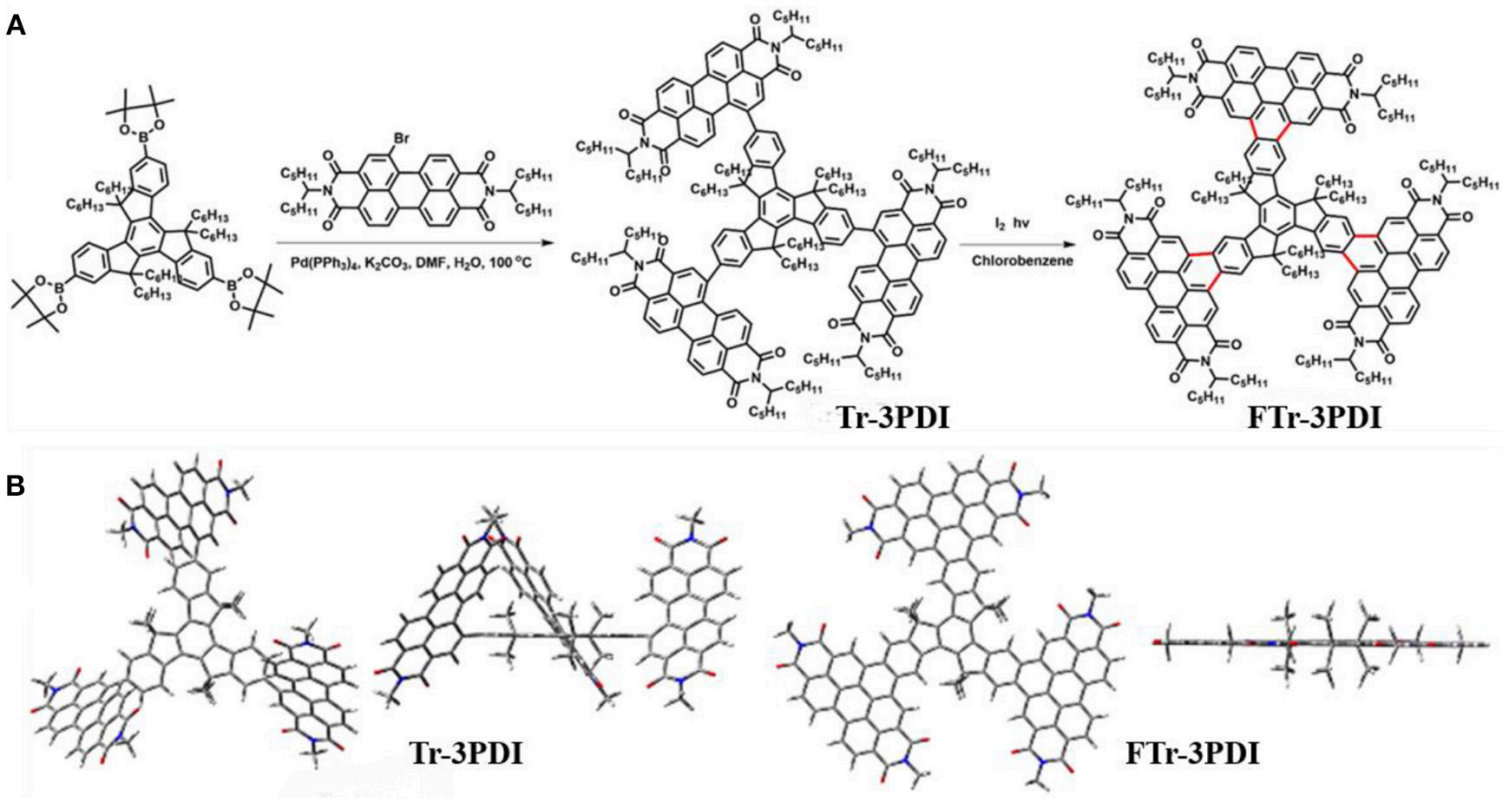
**(A)** Synthetic routes and chemical structures of Tr-3PDI and Fused-Tr-3PDI; **(B)** views of the optimized geometries obtained using DFT calculations at the B3LYP/6-31G(d) level.

Inspired by these achievements, herein, we report the design and synthesis of two star-shaped acceptors named Tr-3PDI and FTr-3PDI, (Scheme 2A) where the truxene core and PDI branches are linked by carbon-carbon single bonds or via ring-fusion, respectively. We further evaluated their potential as electron acceptors in OSCs with poly[4,8-bis(5-(2-ethylhexyl)thiophen-2-yl)benzo[1,2-b;4,5-b]dithiophene-2,6-diyl-*alt*-(4-(2-ethylhexyl)-3-fluorothieno[3,4-b]thiophene)-2-carboxylate-2,6-diyl] (PTB7-Th) as the donor. The solar cells based on Tr-3PDI and FTr-3PDI exhibited a PCE of 2.2 and 3.8%, respectively. The better device performance of the fused acceptor FTr-3PDI is attributable to more favorable energy level alignment with the polymer donor PTB7-Th, more intense light absorption, stronger intermolecular packing, higher electron mobility, and more proper morphology in blend film. This work suggests the potential of ring-fusion strategy for constructing high performance PDI electron acceptors based on truxene core.

## Experimental section

### Materials and synthesis

All reagents were obtained from commercial sources and used without further purification, unless otherwise specified. Scheme 2A shows the synthetic routes of Tr-3PDI and FTr-3PDI. The detailed synthesis procedures are described as following.

#### Tr-3PDI

A mixture of 2,2′,2″-(5,5,10,10,15,15-hexahexyl-10,15-dihydro-5H-diindeno[1,2-a:1′,2′-c]fluorene-2,7,12-triyl)tris(4,4,5,5-tetramethyl-1,3,2-dioxaborolane (truxene boronic acid pinacol ester, 0.613 g, 0.5 mmol) and 5-Bromo-2,9-bis(1-pentylhexyl)anthra[2,1,9-def:6,5,10-d′e′f′]diisoquinoline-1,3,8, 10(2H,9H)-tetrone (monobromo-PDI, 1.746 g, 2.25 mmol) in anhydrous dimethylformamide (40 mL) was degassed for 30 min before Pd(PPh_3_)_4_ (58 mg, 0.05 mmol) and K_2_CO_3_ aqueous solution (2 M, 10 mL) was added. The solution was heated at 95°C for 48 h. Water and dichloromethane were added, and the organic layer was dried over MgSO_4_. After removing the solvent, the crude product was chromatographically purified on silica gel column (eluted with ethyl acetate:petrolem ether = 1:20) to afford Tr-3PDI as a brownish-red solid (0.95 g, 65%). ^1^H NMR (500 MHz, CDCl_3_) δ: 8.74 (m, 18H), 8.60 (m, 6H), 7.62 (m, 6H), 5.25 (m, 6H), 3.04 (m, 6H), 2.17 (m, 30H), 1.29 (m, 112H), 0.86 (m, 72H). ^13^C NMR (125MHz, CDCl_3_) δ: 165.00, 163.91, 155.86, 155.72, 146.31, 142.03, 141.28, 140.62, 138.11, 135.10, 134.57, 132.77, 131.59, 129.98, 129.46, 128.77, 128.34, 127.78, 126.99, 126.80, 123.65, 122.84, 122.26, 56.45, 56.40, 54.95, 54.67, 37.20, 37.07, 32.52, 32.34, 31.91, 31.84, 31.83, 31.81, 31.59, 29.85, 29.80, 29.62, 29.39, 26.78, 26.69, 24.37, 24.13, 22.73, 22.66, 22.64, 22.60, 22.50, 22.43, 14.19,14.16, 14.14. MS (MALDI-TOF) calculated for C_201_H_246_N_6_O_12_, 2938.21; found, 2937.88.

#### FTr-3PDI

Tr-3PDI (293.7 mg, 0.1 mmol) was dissolved in 20 mL chlorobenzene before adding a catalytic amount of iodine (about 2 mg). The resultant mixture was stirred for 1 h under lab environment. After the reactivation process, kept the closed stand-up bottle exposing to irradiation of 500 W mercury lamp for 5 h at room temperature. The color of the solvent turned to brownish-yellow from brownish-red. After the reaction, the solvent was concentrated and the residue was purified by silica gel column chromatography (hexane:dichloromethane = 1:1) to afford a brownish-yellow solid (263.8 mg, 90%). ^1^H NMR (500 MHz, CDCl_3_) δ: 10.65 (m, 9H), 9.67 (s, 3H), 9.37 (m, 6H), 9.17 (m, 6H), 5.62 (m, 6H), 3.83 (m, 6H), 3.17 (m, 6H), 2.63 (m, 12H), 2.10 (m, 12H), 1.47 (m, 90H), 1.38 (m, 15H), 0.91 (m, 80H), 0.36 (m, 18H). ^13^C NMR (125MHz, CDCl_3_) δ: 165.06, 164.52, 155.41, 149.23, 141.71, 139.02, 134.27, 133.92, 129.76, 129.33, 128.73, 127.83, 127.71, 125.41, 125.27, 124.98, 123.50, 123.36, 119.83, 117.66, 57.60, 55.26, 38.26, 32.81, 32.03, 31.56, 29.57, 27.01, 24.91, 22.85, 22.81, 22.32, 14.30, 14.25, 13.84, 13.82. MS (MALDI-TOF) calculated for C_201_H_240_N_6_O_12_, 2932.16; found, 2931.94.

### Instruments and characterization

^1^H and ^13^C NMR spectra were tested on a Bruker AV-500 with tetramethylsilane (TMS) as an internal reference. MALDI-TOF-MS was performed by using a Bruker Agilent1290/maXis impact. UV-vis spectra were measured on a HP 8453 spectrophotometer. Thermogravimetric (TGA) analysis was measured on a NETZSCH TG 209 at a heating rate of 10°C min^−1^ with a nitrogen flow rate of 20 mL min^−1^. Cyclic voltammetry data were measured on a CHI600D electrochemical workstation with Bu_4_NPF_6_ (0.1 M) in acetonitrile as the electrolyte, a carbon electrode and a saturated calomel electrode as the working and reference electrodes, respectively. The thin films were coated on a glassy carbon working electrode. The scan rate was 100 mV s^−1^. The geometry was optimized by Density Functional Theory (DFT) calculations performed at the B3LYP/6-31G(d) level to optimize the ground state geometries of the acceptor molecules using the Gaussian 09. The transient photocurrent of devices was measured by applying 500 nm laser pulses with a pulse width of 120 fs to the devices, which produced a transient voltage signal on a 50 Ω resistor and recorded by an oscilloscope (Tektronix EDS 3052C). The laser pulses were generated from optical parametric amplifier (TOPAS-Prime) pumped by a mode-locked Ti:sapphire oscillator seeded regenerative amplifier with a pulse energy of 1.3 mJ at 800 nm and a repetition rate of 1 KHz (Spectra Physics Spitfire Ace). The atom force microscopy (AFM) images were obtained from a NanoMan VS microscopy under tapping mode. The transmission electron microscopy (TEM) images were characterized with a JEM-2100F instrument.

### Fabrication and characterization of solar cells

The devices of indium tin oxide (ITO)/poly(3,4-ethylenedioxythiophene): poly(styrenesulfonate) (PEDOT:PSS)/ PTB7-Th:acceptor/poly[(9,9-bis(3′-((N,N-dimethyl)-N- ethylammonium)-propyl)-2,7-fluorene)-*alt*-2,7-(9,9-dioctylfluorene)]dibromide (PFN-Br)/Al were fabricated through the following procedures. The ITO-coated glass substrate was cleaned in an ultrasonic bath with deionized water, acetone, and isopropanol, each process was approximately 15 min, and then dried under a stream of dry nitrogen. PEDOT:PSS (Heraeus Clevios PVPA 4083) was spin-coated on top of the above ITO and annealed in air at 150°C for 10 min. Subsequently, the blend solutions of PTB7-Th and truxene-PDI acceptors were prepared by simultaneously dissolving both materials with the optimized weight ratio in ortho-dichlorobenzene and spin-coated on the ITO/PEDOT:PSS electrode (at 1,600 rpm for 60 s) to form an active layer with thickness of about 100 nm. Then PFN-Br and Al layer were thermally deposited onto the active layer through a shadow mask at a vacuum of 5 × 10^−5^ Pa. During the test, an aperture with an area of 3.14 mm^2^ was used. The current density–voltage (*J*–*V*) curves were measured on a computer-controlled Keithley 2400 source meter under 1 sun, the AM 1.5 G spectra came from a class solar simulator (Enlitech, Taiwan), and the light intensity was 100 mW cm^−2^ as calibrated by a China General Certification Center-certified reference monocrystal silicon cell (Enlitech). Before the *J*–*V* measurement, a physical mask with an aperture with precise area of 0.04 cm^2^ was used to define the device area. The external quantum efficiency (EQE) spectra were measured on a commercial QE measurement system (QE-R3011, Enlitech).

### Fabrication and characterization of single-carrier devices

The charge carrier mobilities of PTB7-Th:truxene-PDI acceptor blend films were determined from single-carrier devices with space-charge-limited current (SCLC) model. The device structures of the electron only and hole only devices are ITO/ZnO/PTB7-Th:acceptor/Ca/Al and ITO/PEDOT:PSS/PTB7-Th: acceptor/MoO_3_/Ag respectively. The mobilities were determined by fitting the dark *J*–*V* current to the model of a single carrier SCLC using the equation: *J* = 9ε_0_ε_r_μ*V*^2^/8*d*^3^, where *J* is the current density, *d* is the thickness of the blend films, ε_0_ is the permittivity of free space, ε_r_ is the relative dielectric constant of the transport medium, and μ is the charge carrier mobility. *V* = *V*_app_-*V*_bi_, where *V*_app_ is the applied voltage and *V*_bi_ is the built-in voltage. The carrier mobility can be calculated from the slope of the *J*^1/2^-*V* curves.

## Results and discussion

### Synthesis and characterization

The synthetic routes to Tr-3PDI and FTr-3PDI are shown in Scheme 2A. Tr-3PDI was synthesized via Suzuki cross-coupling reaction between corresponding truxene boronic acid pinacol ester (Lin et al., 2018) and monobromo-PDI (Gao et al., 2017) using Pd(PPh_3_)_4_ as the catalyst. FTr-3PDI was obtained with an excellent yield (90%) from Tr-3PDI by dissolving in chlorobenzene containing a catalytic amount of iodine and exposed to irradiation. Tr-3PDI and FTr-3PDI are characterized by ^1^H NMR, ^13^C NMR, and mass spectra (Figures S1–S6). The optimized geometries of Tr-3PDI and FTr-3PDI are simulated using density functional theoretical (DFT) calculations at the B3LYP/6-31G(d) level (Scheme 2B). Clearly, Tr-3PDI exhibits higher twisted structure with a large dihedral angle over 50° owing to the steric hindrance effect. After the oxidative ring-fusion, each PDI moiety is tethered to truxene through benzene rings, resulting in an overall planarity structure because of the high rigidity and coplanarity of truxene core. Both acceptors are soluble in common organic solvents such as dichloromethane, chloroform, chlorobenzene, and ortho-dichlorobenzene at room temperature. The reason is that there are six hexyl chains on the truxene core, providing outstanding solubility for the resulting compounds.

The thermal properties of Tr-3PDI and FTr-3PDI were analyzed by thermogravimetric analysis (TGA) and differential scanning calorimetry (DSC). As shown in Figure S7, both truxene-PDI acceptors have decomposition temperature with 5% weight loss above 400°C. Moreover, there is no clear phase transition in DSC curves, which is indicative of the amorphous nature of Tr-3PDI and FTr-3PDI.

**Figure 1:**
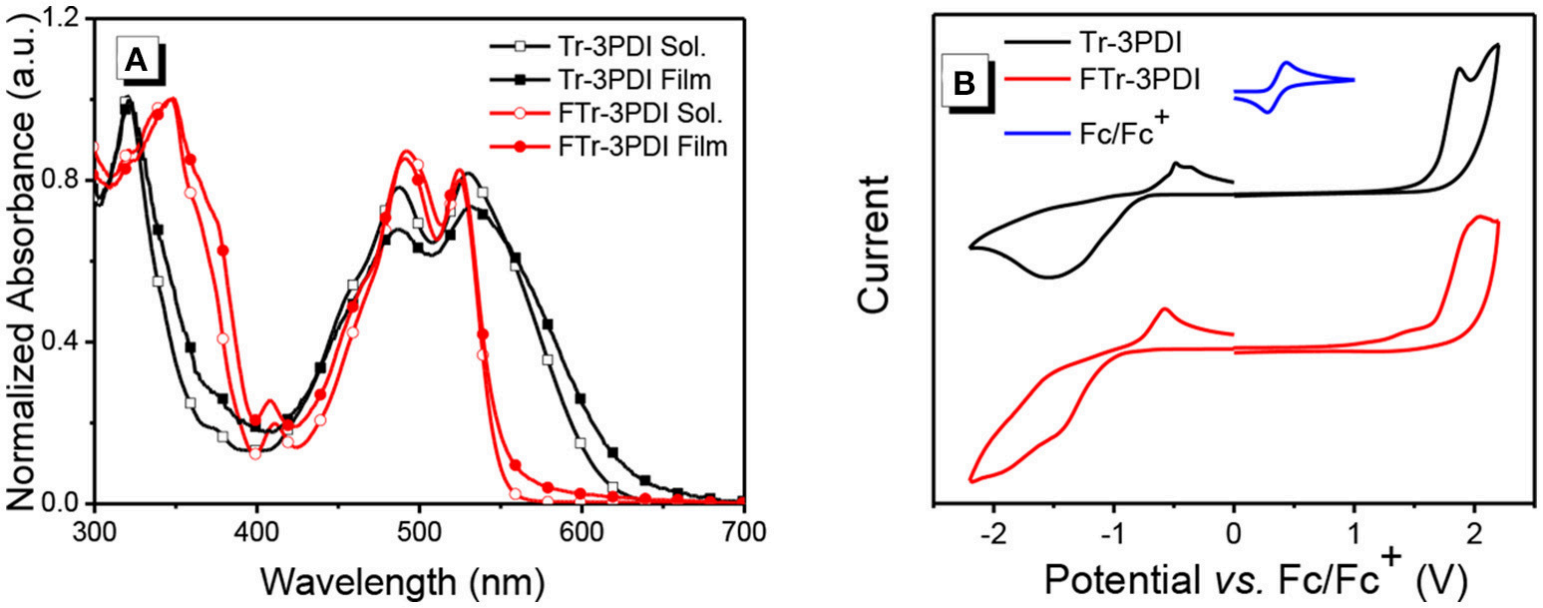
**(A)** UV-vis absorption spectra of Tr-3PDI and FTr-3PDI in chloroform solutions and as thin films; **(B)** cyclic voltammograms of Tr-3PDI and FTr-3PDI.

The UV-vis absorption spectra of the two acceptors in solutions and as thin films are shown in Figure 1A, Figure S8, and the relevant data are summarized in Table 1. Both Tr-3PDI and FTr-3PDI show two absorption bands with one in the short wavelength region of 300–400 nm and one in the longer wavelength region of 400–600 nm. The intense absorption in the short wavelength region is attributable to the large coplanar core of truxene. FTr-3PDI shows little difference in normalized absorption spectra from the solution state to the film state, while the solid state Tr-3PDI has extended and redshifted absorption compared to the solution state. Notably, although the two compounds have very similar absorption maxima in both solution and solid state, FTr-3PDI shows considerably blue-shifted absorption onset as compared to Tr-3PDI, which could be related to the reduced conformational disorder via ring-fusion and then weakens the intramolecular charge transfer between truxene and PDI moieties. In addition, FTr-3PDI exhibits higher absorption coefficient than Tr-3PDI (Figure S8). The optical band gaps (*E*_g_) are calculated to be 1.96 eV for Tr-3PDI, and 2.23 eV for FTr-3PDI (Table 1).

**Table 1:** Optical and electrochemical properties of Tr-3PDI and FTr-3PDI.

**Acceptors**	**$\lambda_{\text{onset}}^{\text{film}}$ (nm)**	**$E_{\text{g}}^{\text{opt a}}$ (eV)**	**$E_{\text{HOMO}}^{\text{b}}$ (eV)**	**$E_{\text{LUMO}}^{\text{c}}$ (eV)**
Tr-3PDI	633	1.96	−6.09	−3.64
FTr-3PDI	556	2.23	−6.11	−3.44

a *Calculated from $E_{\text{g}}^{\text{opt}}$ = 1240/$\lambda_{\text{onset}}^{\text{film}}$ eV; ^b^Calculated from E_HOMO_ = –e($E_{\text{ox}}^{\text{onse}}$-E_Fc/Fc+_+4.8) eV; ^c^Calculated from E_LUMO_ = –e($E_{\text{red}}^{\text{onset}}$-E_Fc/Fc+_+4.8) eV*.

The energy levels of the acceptors were determined by cyclic voltammetry (CV) experiments. The half-wave potential of Fc/Fc^+^ was measured to be 0.36 V, and the energy levels of the highest occupied molecular orbital (HOMO) and lowest unoccupied molecular orbital (LUMO) were estimated from the onset oxidation ($E_{\text{ox}}^{\text{onset}}$) and reduction ($E_{\text{red}}^{\text{onset}}$) potentials by equations: *E*_HOMO_ = –*e*($E_{\text{ox}}^{\text{onset}}$-*E*_Fc/Fc+_+4.8) and *E*_LUMO_ = –*e*($E_{\text{red}}^{\text{onset}}$-*E*_Fc/Fc+_+4.8), respectively (Li et al., 1999). The CV curves are shown in Figure 1B, and the relevant data are listed in Table 1. The HOMO/LUMO levels are −6.09/−3.64 eV for Tr-3PDI and −6.11/−3.44 eV for FTr-3PDI, respectively. The slightly up-shifted LUMO level of FTr-3PDI will help to offer a higher open-circuit voltage (*V*_oc_), and the down-shifted HOMO level is favorable for hole transfer from excited acceptor phase to donor phase in BHJ OSCs (Duan et al., 2016a, 2017b, 2018; Jia et al., 2017).

### Photovoltaic properties

**Figure 2:**
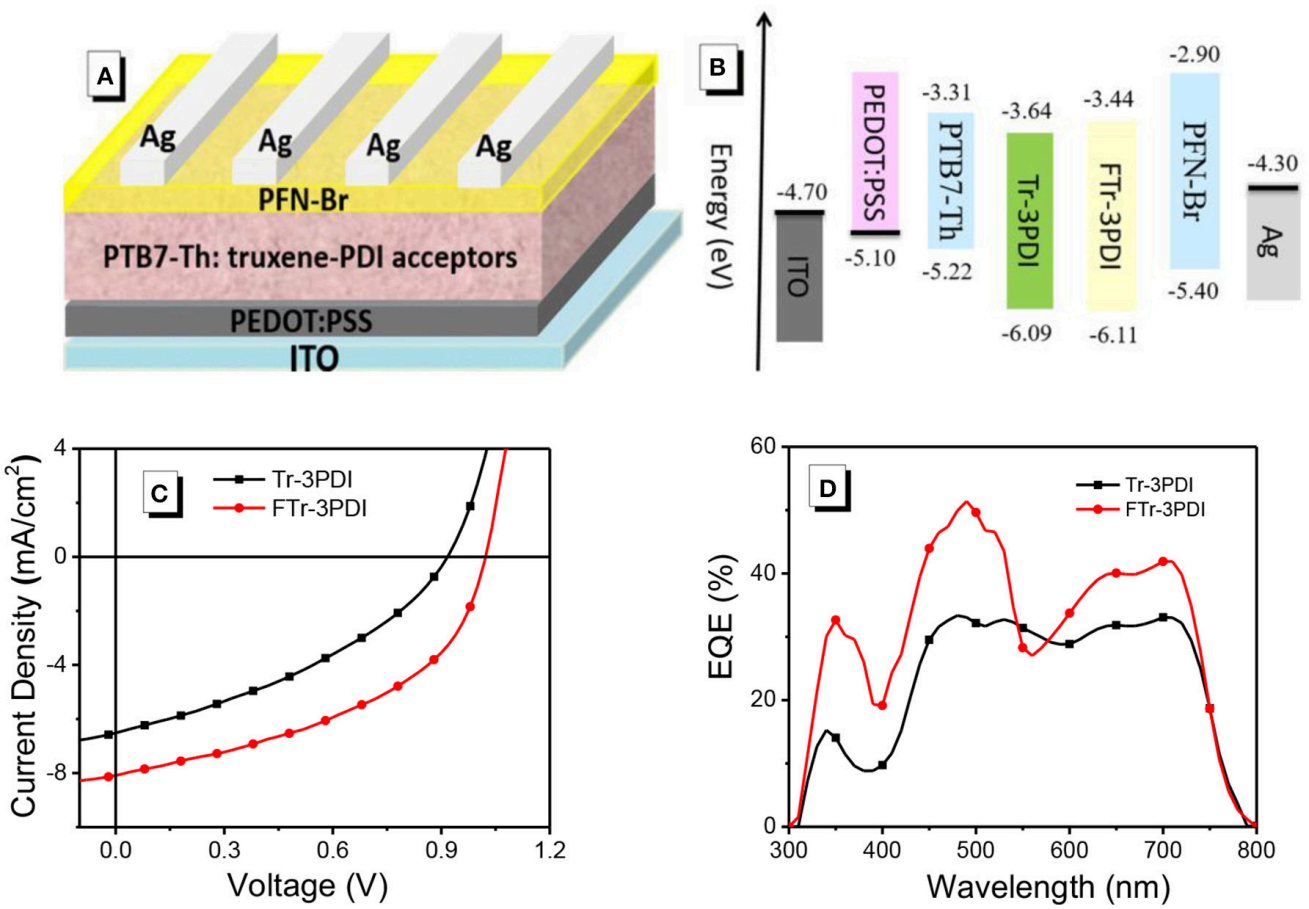
**(A)** The device structure; **(B)** energy-level diagrams for all the materials used in this research; **(C)** *J–V* characteristics; and **(D)** EQE spectra of the OSCs based on PTB7-Th and truxene-PDI acceptors.

The photovoltaic properties of Tr-3PDI and FTr-3PDI were evaluated in OSCs under AM1.5G illumination at 100 mW cm^−2^ with a device structure of ITO/PEDOT:PSS/PTB7-Th:acceptor/PFN-Br/Ag (Figure 2A). PTB7-Th was used as the donor because of its strong optical absorption at long-wavelength region (Figure S9), which can achieve complementary absorption with our truxene-based acceptors (Zhang et al., 2015; McAfee et al., 2017; Welsh et al., 2018). The schematic energy diagram of individual components is displayed in Figure 2B, suggesting proper energy level alignment of each layer in the device. The devices were fully optimized in terms of host solvent, donor/acceptor weight ratios, active layer thickness, solvent additives, thermal annealing, and solvent annealing. The current density–voltage (*J–V*) curves of the optimized devices are shown in Figure 2C, and the photovoltaic parameters are summarized in Table 1. The device parameters under various conditions are collected in Tables S1–S8. The optimized device of Tr-3PDI afforded a PCE of 2.2% along with a *V*_oc_ of 0.92 V, a short-circuit current density (*J*_sc_) of 6.5 mA cm^−2^, and a fill factor (FF) of 0.37. The ring-fused acceptor FTr-3PDI produced a higher PCE of 3.8% along with a *V*_oc_ of 1.02 V, a *J*_sc_ of 8.1 mA cm^−2^, and an FF of 0.46 (Table 2). The higher *V*_oc_ of FTr-3PDI is consistent with the up-shifted LUMO level. The difference in *J*_sc_ of the solar cells can be explained by their external quantum efficiency (EQE) spectra (Figure 2D) (Duan et al., 2016b; Wu et al., 2016). The PTB7-Th:FTr-3PDI blend film show higher EQE than PTB7-Th:Tr-3PDI almost in the whole spectral range of 300–800 nm, which is because of the enhanced light absorption of the former and more efficient charge generation. The PTB7-Th:FTr-3PDI device also shows higher FF than the PTB7-Th:Tr-3PDI device, suggesting improved charge transport, reduced charge recombination, and more optimal phase separated morphology (Duan et al., 2011; Xie et al., 2012).

**Table 2:** Photovoltaic parameters of OSCs based on PTB7-Th and truxene-PDI acceptors under AM1.5G illumination at 100 mW cm^−2^.

**Acceptor devices**	***V*_oc_ (V)**	***J*_sc_ (mA cm^−2^)**	**FF**	**PCE (%)**
PTB7-Th: Tr-3PDI	0.92	6.5	0.37	2.2
PTB7-Th: FTr-3PDI	1.02	8.1	0.46	3.8

### Charge transport and recombination

The charge transport were investigated in single-carrier devices with a device structure of ITO/ZnO/ active layer /Ca/Al for electron only devices and ITO/PEDOT:PSS/ Active layer /MoO_3_/Ag for hole only devices, respectively. The electron and hole mobilities were acquired by fitting the *J*–*V* with space-charge-limited current (SCLC) model. The *J*–*V* curves of the devices for pure acceptors and blend films are shown in Figures S10, S11. As shown in Table 3, the FTr-3PDI pure film exhibits higher electron mobility (μ_e_) of 2.4 × 10^−6^ cm^2^ V^−1^ s^−1^ than Tr-3PDI film (3.2 × 10^−7^ cm^2^ V^−1^ s^−1^), which support that the ring-fusion strategy is successful. As for blend films, the hole mobilities (μ_h_) were estimated to be 1.2 × 10^−3^ cm^2^ V^−1^ s^−1^ for PTB7-Th:Tr-3PDI and 8.2 × 10^−3^ cm^2^ V^−1^ s^−1^ for PTB7-Th:FTr-3PDI, which are comparable with the value that obtained from PTB7-Th:fullerene devices (Huang et al., 2016). In contrast, theμ_e_ of the blend films of PTB7-Th:truxene-PDI acceptors were measured to be 5.8 × 10^−6^ cm^2^ V^−1^ s^−1^ for PTB7-Th:Tr-3PDI and 1.3 × 10^−5^ cm^2^ V^−1^ s^−1^ for PTB7-Th:FTr-3PDI, which are more than two orders of magnitude lower than that of PTB7-Th:fullerene film (Lin et al., 2015). The low electron mobility and highly imbalanced μ_e_/μ_h_ seriously obstruct the charge transport and resulted in more bimolecular recombination, which in turn led to low FF and *J*_sc_. For the solar cells with very imbalanced μ_e_/μ_h_, the device performance will be determined by the slower charge carrier, which is electron in these cases. The higher electron mobility in PTB7-Th:FTr-3PDI will thus result in better device performance.

**Table 3:** The relevant parameters related to charge transport and recombination of PTB7-Th: truxene-PDI acceptor devices.

**Active layer**	**μ_h_ (cm^2^ V^−1^ s^−1^)**	**μ_e_ (cm^2^ V^−1^ s^−1^)**	**S**	**α**	**Charge-extraction time (μs)**	**Charge carrier lifetime (μs)**
Tr-3PDI	–	3.2 × 10^−7^	–	–	–	–
FTr-3PDI	–	2.4 × 10^−6^	–	–	–	–
PTB7-Th:Tr-3PDI	1.2 × 10^−3^	5.8 × 10^−6^	0.88	2.1	0.15	9.72
PTB7-Th:FTr-3PDI	8.2 × 10^−3^	1.3 × 10^−5^	0.89	1.9	0.16	15.31

**Figure 3:**
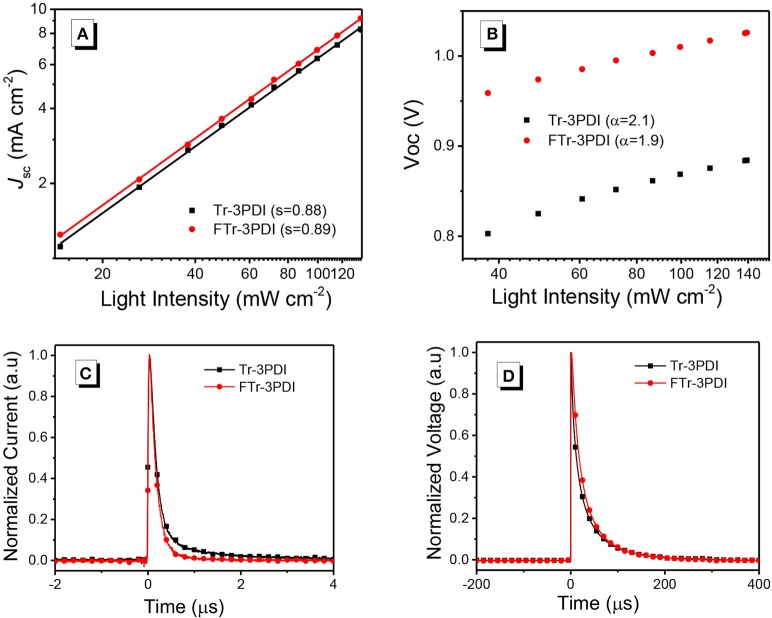
**(A)** Current density vs. light intensity characteristics, and **(B)** open-circuit voltage vs. natural logarithm of light intensity characteristics for devices based on PTB7-Th and truxene-PDI acceptors; **(C)** transient photocurrent measurements; and **(D)** transient photovoltage of the relevant OSC devices.

To study the charge-recombination of these devices, we investigated the photocurrent (*J*_sc_) as a function of light intensity (*P*_in_, from 1 to 100 mW cm^−2^), with the relevant characteristics plotted in Figure 3A. Generally, *J*_sc_ and *P*_in_ follow the relationship of *J*_sc_∝$P_{\text{in}}^{\text{S}}$. If all free carriers are swept out and collected at the electrodes prior to recombination, the slope (S) should be equal to 1, while *S* < 1 indicates some extent of bimolecular recombination (Kyaw et al., 2013). The *S*-values of the two devices are 0.89 for PTB7-Th:FTr-3PDI and 0.88 for PTB7-Th:Tr-3PDI, respectively, which indicates the existence of some extent of bimolecular recombination. The charge recombination mechanism of the truxene-PDI-based OSCs are also investigated by estimating the slope (α) of *V*_oc_ vs. ln*P* (*P* is light intensity). In principle, the slope α approaching *k*_B_*T*/*q* implies that the device has only bimolecular recombination, and the slope α approaching 2*k*_B_*T*/*q* suggests that the monomolecular recombination or trap-assisted recombination dominates in OSCs (where *T, k*_B_, and *q* are the Kelvin temperature, Boltzmann constant, and elementary charge, respectively) (Koster et al., 2005; Lu et al., 2015). The α values for PTB7-Th:Tr-3PDI and PTB7-Th:FTr-3PDI are 2.1 and 1.9, respectively, which indicate considerable monomolecular recombination or trap-based recombination in these devices (Figure 3B and Table 3).

Transient photocurrent (TPC) and transient photovoltage (TPV) measurements were used to study the charge recombination dynamics and charge-extraction process in OSCs. From TPC analysis (Figure 3C), the charge-extraction time of the PTB7-Th:FTr-3PDI based device (0.15 μs) is slightly shorter than the PTB7-Th:Tr-3PDI based device (0.16 μs), suggesting increased charge extraction rate (Jin et al., 2016). From TPV analysis (Figure 3D), the charge carrier lifetime increased from 9.72 μs for the PTB7-Th:Tr-3PDI device to 15.31 μs for the PTB7-Th:FTr-3PDI device (Table 3), indicating reduced recombination loss for the PTB7-Th:FTr-3PDI device (Shuttle et al., 2008). The increased charge extraction rate and longer carrier lifetime thus explained the improved FF value and the higher PCE of the PTB7-Th:FTr-3PDI device.

### Morphology characterization

**Figure 4:**
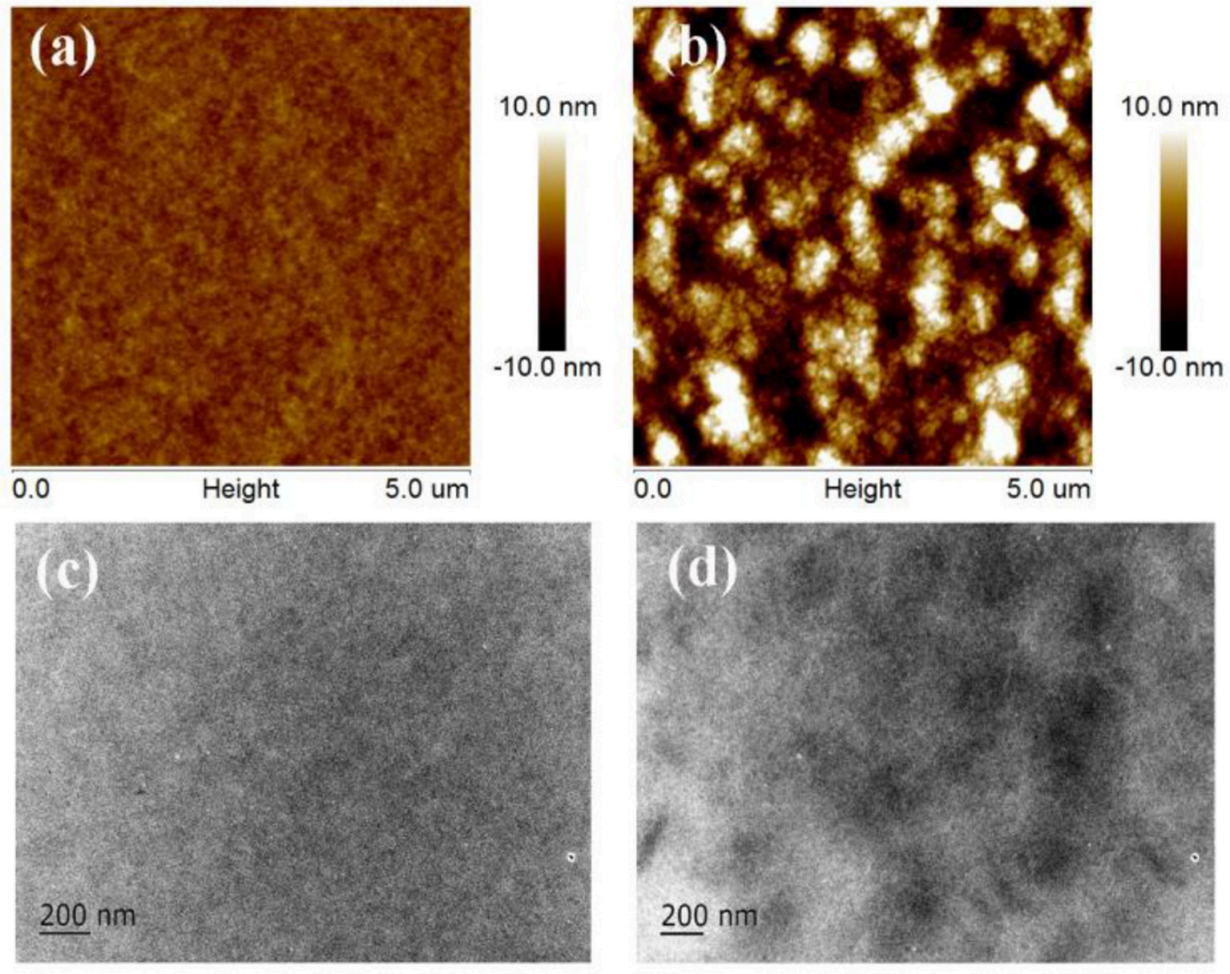
AFM and TEM images of the blend films of PTB7-Th:truxene-PDI acceptor: **(A,C)** PTB7-Th:Tr-3PDI, **(B,D)** PTB7-Th:FTr-3PDI.

The morphology of the active layers was studied by atom force microscopy (AFM) and transmission electron microscopy (TEM). The AFM images and TEM images of the blend films are shown in Figure 4. The blend film of PTB7-Th:Tr-3PDI (Figures 4A,C) is homogeneous with a root-mean-square (RMS) surface roughness of 0.72 nm. The uniform film reveals intimately mixed blends without noteworthy phase separation (Duan et al., 2017c; Wang J. Y. et al., 2017; Wen et al., 2018). With such a morphology, charge transport is impeded. The film of PTB7-Th:FTr-3PDI exhibits obvious phase separation with granulate features (Figures 4B,D), resulting in a relative coarse surface with a RMS surface roughness of 3.88 nm. The less phase-separated morphology of PTB7-Th:Tr-3PDI film could be a reason of the enhanced charge recombination and imbalanced hole/electron transport, which is in accordance with the analysis demonstrated above based on TPC, TPV, and charge carrier mobility measurements.

## Conclusion

In summary, two electron acceptors with a truxene core and three PDIs branches linked by carbon-carbon single bonds (Tr-3PDI) or adjoin benzene ring (FTr-3PDI) are designed and developed. The FTr-3PDI shows up-shifted energy levels, enhanced absorption, improved charge mobility, and more favorable morphology as compared to Tr-3PDI. These merits further lead to higher *V*_oc_, *J*_sc_, and FF in resulting OSCs, respectively. The OSCs of PTB7-Th:FTr-3PDI blend shows a PCE of 3.8%, which is almost two times higher than that of PTB7-Th:Tr-3PDI blend. This work demonstrates a successful construction of star-shaped non-fullerene electron acceptor materials based on a truxene core and multiple PDI branches via ring-fusion to improve the performance of OSCs.

## Author contributions

KL, FH and YC: Designed experiments; KL, SW, ZW, QY, XL, and XJ: Carried out experiments; JJ, XfJ, and PL: Analyzed experimental results; KL and CD: Wrote the manuscript.

### Conflict of interest statement

The authors declare that the research was conducted in the absence of any commercial or financial relationships that could be construed as a potential conflict of interest.
