# Supplementary material for: Electron Acceptors With a Truxene Core and Perylene Diimide Branches for Organic Solar Cells: The Effect of Ring-Fusion
Source: Front Chem. 2018 Sep 4;6:328. doi: 10.3389/fchem.2018.00328 (PMC6131300; doi:10.3389/fchem.2018.00328)
Supplement: Supplementary file 1 [file Data_Sheet_1.PDF]

Electron acceptors with a truxene core and perylene diimide branches for organic solar cells: the effect of ring-fusion

Kaiwen Lin‡, Shiliang Wang‡, Zhenfeng Wang, Qingwu Yin, Xi Liu, Jianchao Jia, Xiao'e Jia, Peng Luo, Xiaofang Jiang, Chunhui Duan*, Fei Huang* and Yong Cao

‡*These authors contributed equally to this work.*

**Corresponding author. Institute of Polymer Optoelectronic Materials and Devices, State Key Laboratory of Luminescent Materials and Devices, South China University of Technology, Guangzhou 510640, P. R. China.*

E-mail: duanchunhui@scut.edu.cn; msfhuang@scut.edu.cn

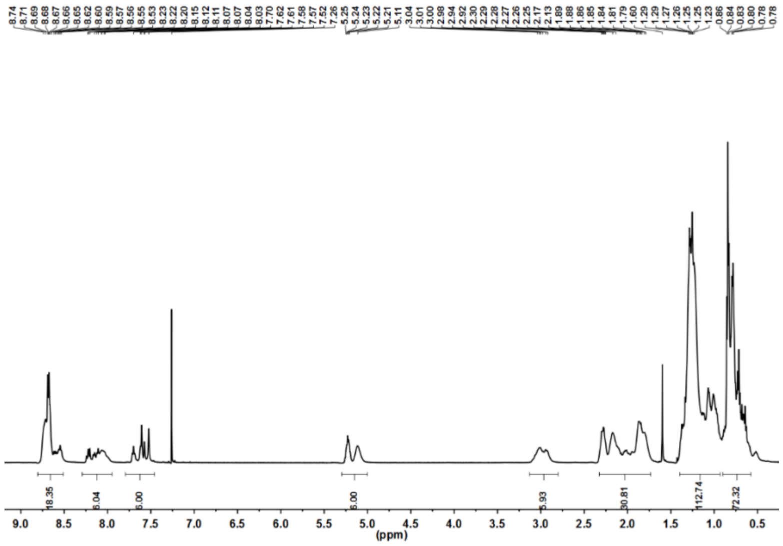

FIGURE S1 ^{1}H NMR spectrum of Tr-3PDI.

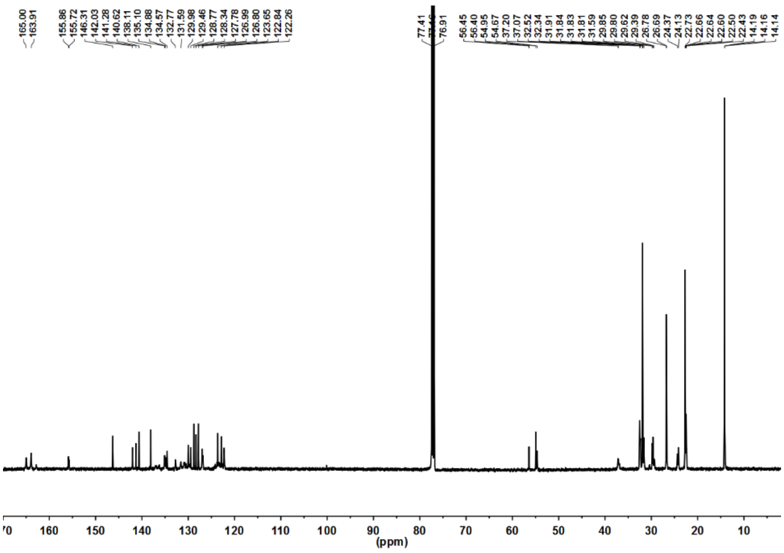

FIGURE S2 ^{13}C NMR spectrum of Tr-3PDI.

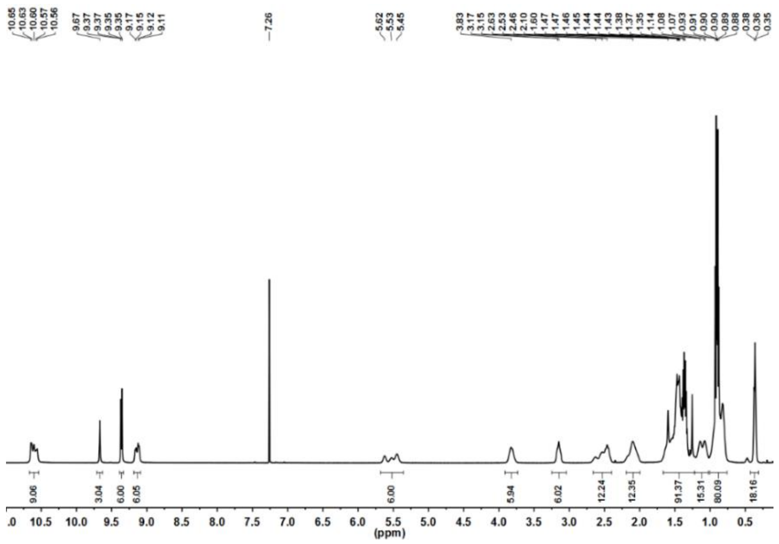

FIGURE S3 ^{1}H NMR spectrum of FTr-3PDI.

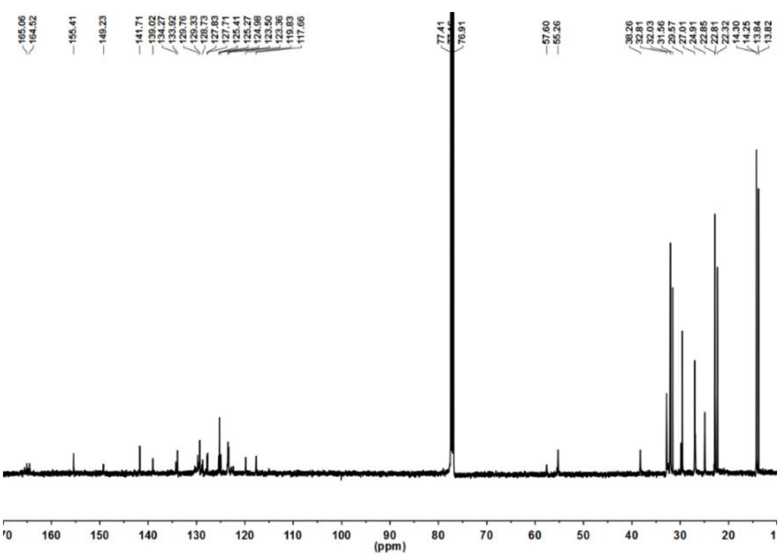

FIGURE S4 ^{13}C NMR spectrum of FTr-3PDI.

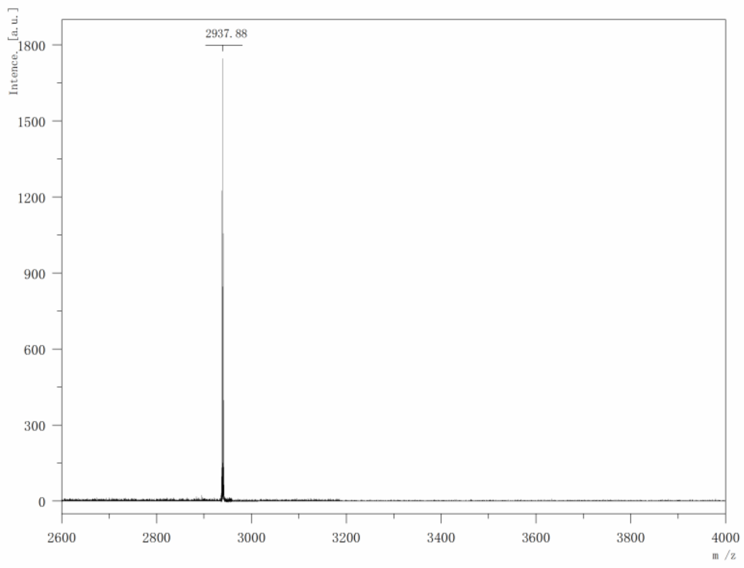

FIGURE S5 Maldi-TOF of Tr-3PDI.

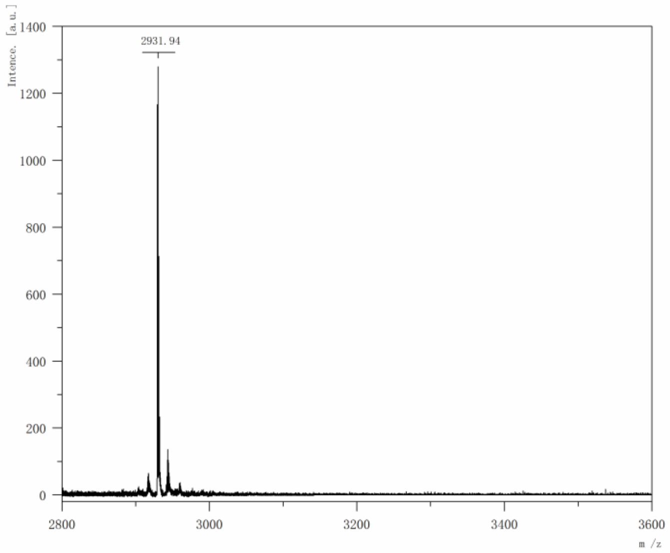

FIGURE S6 Maldi-TOF of FTr-3PDI.

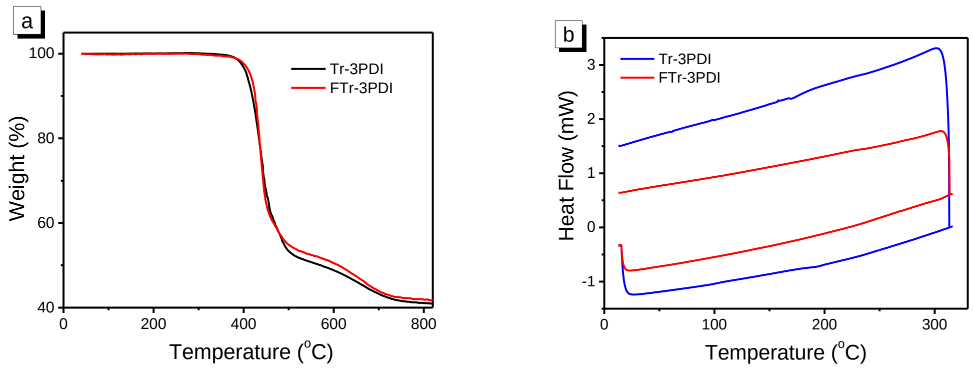

FIGURE S7 (a) TGA and (b) DSC spectra of Tr-3PDI and FTr-3PDI.

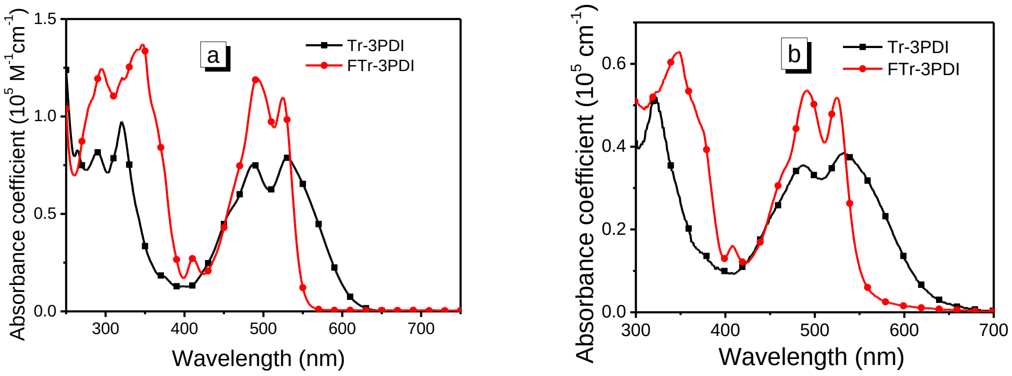

FIGURE S8 UV-vis absorption spectra of Tr-3PDI and FTr-3PDI in chloroform solution (1.0×10^{-5} M) (a) and as thin film (b).

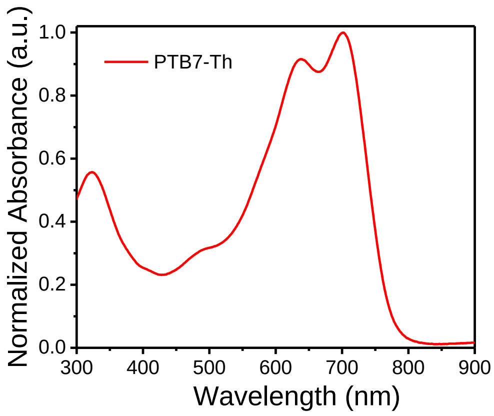

FIGURE S9 UV-vis absorption spectra of PTB7-Th.

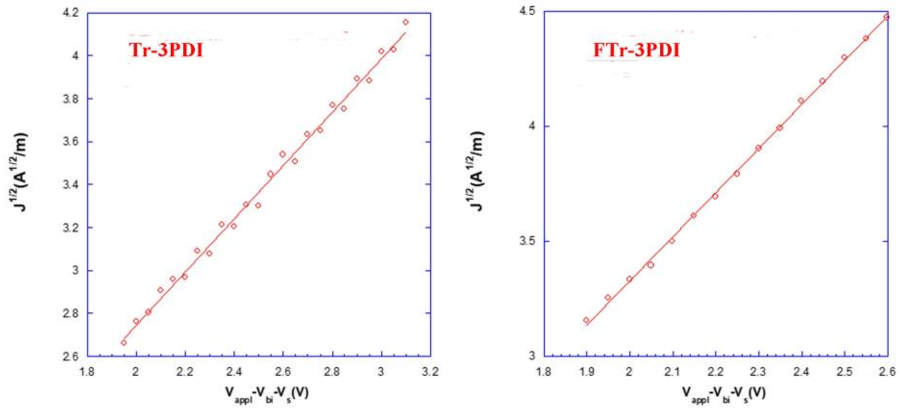

FIGURE S10 *J–V* characteristics of the pure truxene-PDI acceptors in electron-only devices.

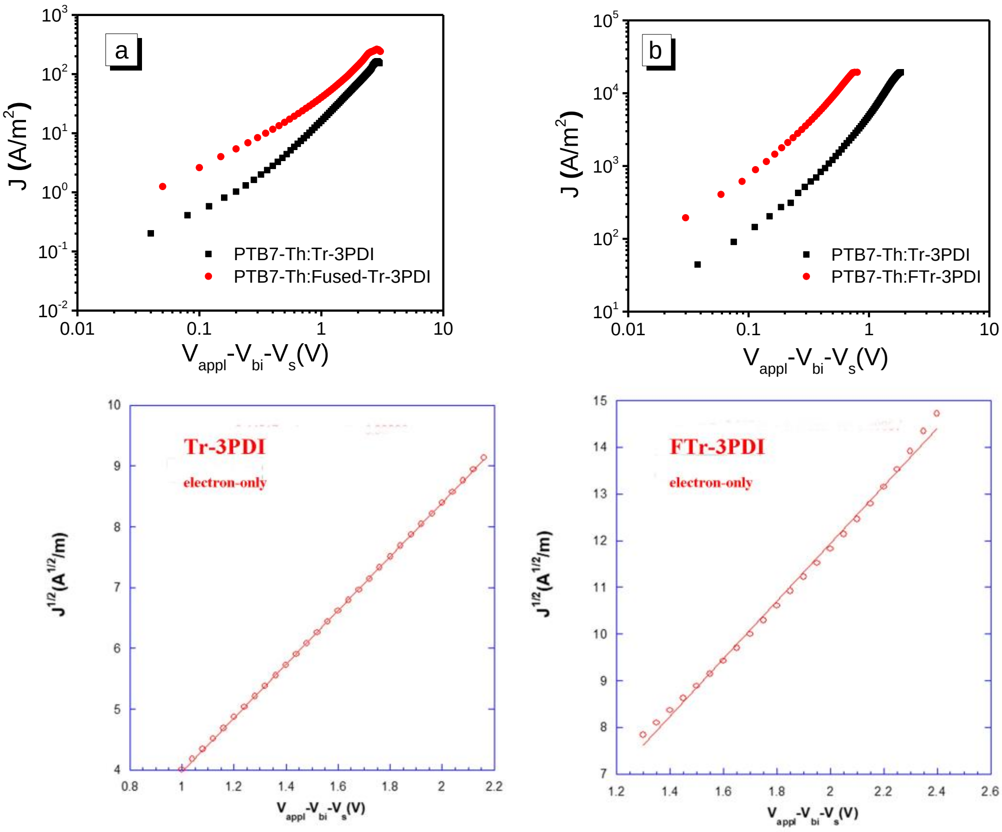

FIGURE S11 *J–V* characteristics of the blend films of PTB7-Th:truxene-PDI acceptors in electron-only devices (a) and hole-only devices (b).

TABLE S1 The optimization of host solvents (the thickness of active layers is 90 nm±5nm; donor/acceptor weight ratios is 1:1).

Sample	solvent	V_{oc} (V)	J_{sc} (mA cm^{-2})	J_{sc} (mA cm^{-2}) (%)	PCE (%)
PTB7-Th:Tr-3PDI	CF	0.89	4.65	32.78	1.36
	**o-DCB**	0.90	6.18	34.81	**1.95**
	CB	0.89	6.00	32.78	1.74
PTB7-Th: FTr-3PDI	CF	0.99	6.22	35.29	2.17
	**o-DCB**	1.02	5.91	44.75	**2.65**
	CB	1.02	5.44	45.12	2.50

TABLE S2 The optimization of donor/acceptor (D/A) weight ratios (the host solvents is o-DCB; the thickness of active layers is 90 nm±5nm).

Sample	D/A Ratio	V_{oc} (V)	J_{sc} (mA cm^{-2})	FF (%)	PCE (%)
PTB7-Th:Tr-3PDI	1:1	0.89	5.89	34.96	1.88
	**1:1.5**	**0.89**	**6.14**	**35.12**	**1.96**
	1:2	0.89	6.13	34.83	1.91
	1:2.5	0.89	5.92	34.5	1.82
PTB7-Th: FTr-3PDI	1:1	1.03	4.48	45.21	2.49
	1:1.5	1.02	5.78	42.81	2.52
	**1:2**	**1.02**	**5.91**	**45.75**	**2.85**
	1:2.5	1.02	5.58	43.56	2.44

TABLE S3 The optimization of thickness for PTB7-Th:Tr-3PDI (the host solvents is o-DCB; D/A) weight ratio is 1:1.5).

Thickness(nm)	V_{oc} (V)	J_{sc} (mA cm^{-2})	FF (%)	PCE (%)
162	0.88	4.96	33.21	1.45
152	0.88	5.03	32.78	1.46
138	0.86	5.42	31.45	1.47
120	0.90	5.61	34.00	1.72
113	0.90	5.99	35.18	1.91
**102**	**0.91**	**5.89**	**37.21**	**1.96**
92	0.91	5.68	37.42	1.93
76	0.90	4.90	38.52	1.69

TABLE S4 The optimization of thickness for PTB7-Th:FTr-3PDI (the host solvents is o-DCB; D/A) weight ratio is 1:2).

Thickness(nm)	V_{oc} (V)	J_{sc} (mA cm^{-2})	FF (%)	PCE (%)
49	0.98	3.38	34.91	1.16
68	1.00	5.46	39.63	2.17
76	1.01	5.91	40.94	2.43
85	1.02	5.81	42.19	2.71
**96**	**1.01**	**7.16**	**44.17**	**3.20**
112	1.02	5.54	42.1	2.45
125	1.02	4.88	41.95	2.09
136	1.02	4.62	43.78	2.07
150	1.00	4.12	40.30	1.66

TABLE S5 The optimization of thermal annealing (TA) for PTB7-Th:FTr-3PDI (the host solvents is o-DCB; D/A weight ratio is 1:2; the thickness of active layers is 100 nm±5nm).

Sample	V_{oc} (V)	J_{sc} (mA cm^{-2})	FF (%)	PCE (%)
N/A	0.96	7.00	37.53	2.53
	1.01	6.55	41.89	2.78
80℃ 5min	1.00	6.87	42.47	2.88
	1.01	6.55	40.41	2.67
100℃ 5min	1.01	5.99	41.35	2.51
	1.02	5.60	43.81	2.51
**120℃ 5min**	**1.02**	**6.91**	**44.07**	**3.05**
	**1.03**	**7.15**	**45.12**	**3.33**
140℃ 5min	1.01	6.63	40.61	2.73
	0.98	6.63	37.19	2.41

TABLE S6 The optimization of subsequent solvent annealing (SVA) for PTB7-Th:FTr-3PDI (the host solvents is o-DCB; D/A weight ratio is 1:2; the thickness of active layers is 100 nm±5nm).

Sample	V_{oc} (V)	J_{sc} (mA cm^{-2})	FF (%)	PCE (%)
N/A	1.02	5.41	41.83	2.31
	1.02	5.64	41.98	2.41
860rpm CF 15s	1.00	5.46	39.93	2.17
**860rpm CF 30s**	**1.03**	**5.82**	**44.07**	**2.63**
860rpm CF 1min	1.01	5.95	41.90	2.53
860rpm THF 15s	1.03	4.84	45.04	2.26
860rpm THF 30s	1.01	5.99	40.24	2.43
**860rpm THF 1min**	**1.02**	**6.04**	**43.67**	**2.70**
860rpm N/A	1.02	5.79	43.10	2.55

TABLE S7 The optimization of solvent additives (the host solvents is o-DCB; D/A weight ratio is 1:1.5 for PTB7-Th:Tr-3PDI and 1:2 for PTB7-Th:FTr-3PDI; the thickness of active layers is 100 nm±5nm).

Sample	solvent additives	V_{oc} (V)	J_{sc} (mA cm^{-2})	FF (%)	PCE (%)
PTB7-Th:Tr-3PDI	2%DIO	0.93	5.40	37.93	1.90
	**2%CN**	**0.92**	**6.51**	**36.51**	**2.18**
	1%DIO&1%CN	0.93	5.47	37.84	1.92
PTB7-Th: FTr-3PDI	2%DIO	1.06	4.52	47.91	2.29
	**2%CN**	**1.02**	**8.09**	**45.63**	**3.77**
	1%DIO&1%CN	1.02	6.10	49.53	3.07

TABLE S8 The optimization of solvent additives (CN) (the host solvents is o-DCB; D/A weight ratio is 1:1.5 for PTB7-Th:Tr-3PDI and 1:2 for PTB7-Th:FTr-3PDI; the thickness of active layers is 100 nm±5nm).

Sample	solvent additives	V_{oc} (V)	J_{sc} (mA cm^{-2})	FF (%)	PCE (%)
PTB7-Th:Tr-3PDI	1% CN	0.91	6.36	37.23	2.15
	**2%CN**	**0.92**	**6.51**	**36.51**	**2.18**
	3%CN	0.93	5.40	37.93	1.90
PTB7-Th: FTr-3PDI	1%CN	1.03	7.15	45.10	3.32
	**2%CN**	**1.02**	**8.09**	**45.63**	**3.77**
	3%CN	1.02	6.10	49.53	3.07
